# Cyclophilin E (CypE) Functions as a Positive Regulator in Osteoblast Differentiation by Regulating the Transcriptional Activity of Runx2

**DOI:** 10.3390/cells12212549

**Published:** 2023-10-31

**Authors:** Meiyu Piao, Sung Ho Lee, Yuankuan Li, Joong-Kook Choi, Chang-Yeol Yeo, Kwang Youl Lee

**Affiliations:** 1 College of Pharmacy, Research Institute of Pharmaceutical Sciences, Chonnam National University, Gwangju 61186, Republic of Korea; my101park@gmail.com (M.P.); puzim23@gmail.com (S.H.L.); lyk208943@jnu.ac.kr (Y.L.); 2 Division of Biochemistry, College of Medicine, Chungbuk National University, Cheong-Ju 28644, Republic of Korea; jkchoi@chungbuk.ac.kr; 3 Department of Life Science and Research Center for Cellular Homeostasis, Ewha Woman’s University, Seoul 03760, Republic of Korea

**Keywords:** CypE, Runx2, osteoblast differentiation

## Abstract

Cyclophilin E (CypE) belongs to the cyclophilin family and exhibits peptidyl-prolyl *cis-trans* isomerase (PPIase) activity. It participates in various biological processes through the regulation of peptidyl-prolyl isomerization. However, the specific role of CypE in osteoblast differentiation has not yet been elucidated. In this study, we first discovered the positive impact of CypE on osteoblast differentiation through gain or loss of function experiments. Mechanistically, CypE enhances the transcriptional activity of Runx2 through its PPIase activity. Furthermore, we identified the involvement of the Akt signaling pathway in CypE’s function in osteoblast differentiation. Taken together, our findings indicate that CypE plays an important role in osteoblast differentiation as a positive regulator by increasing the transcriptional activity of Runx2.

## 1. Introduction

In recent years, osteoporosis has emerged as a disease that significantly impairs public health. It is characterized by the loss of bone mass and systemic damage to the microstructure, resulting in an increased risk of fractures [1]. Currently, the treatment of osteoporosis is divided into two approaches: anti-resorptive drugs, which decrease bone resorption, and anabolic drugs, which stimulate bone formation [2,3]. Despite the efforts to optimize the timing and dosage of administration, most of these drugs have limitations and can cause partial side effects [4,5]. Therefore, finding effective and long-term drugs for the treatment of osteoporosis remains a challenge.

Up to now, the pathogenesis of osteoporosis has been well elucidated with the underlying cellular and molecular processes [6,7]. At the cellular level, dynamic stabilization and cellular communication between osteoblasts and osteoclasts maintain bone homeostasis. This dynamic process is also known as bone remodeling [8]. The disruption of bone remodeling leads to osteoporosis. Therefore, inducing osteoblast differentiation is an important process to stimulate bone formation. Various signaling pathways, cytokines, and transcription factors are involved in regulating osteoblast differentiation [9]. In particular, the transcription factor Runx2 has been shown to play a crucial role in the induction of osteoblast differentiation and bone formation [10,11]. Runx2 belongs to the family of Runt-related transcription factors, which contain the Runt domain [12]. Previous studies have shown that Runx2 regulates the expression of osteogenic genes by binding to target genes through its Runt domain [13]. Moreover, studies on *Runx2*-null mice studies revealed the essential biological roles of Runx2 in osteoblast differentiation and bone formation [14,15]. Undoubtedly, regulating the expression and transcriptional activity of Runx2 is a major target for affecting osteoblast differentiation.

Cyclophilin E (CypE), also known as Cyp33, is a member of the cyclophilin family and exhibits peptidyl-prolyl *cis-trans* isomerase (PPIase) activity [16]. It shares similarities with other cyclophilins such as cyclophilin A (CypA), cyclophilin B (CypB), cyclophilin D (CypD), and Pin1, which are involved in protein folding, trafficking, and signal transduction [17]. CypE has a cyclophilin-like domain at the C-terminus and an RNA recognition motif at the N-terminus [18]. The C-terminus of CypE shows homology to CypA in terms of the amino acid sequence [18], suggesting that the functions of CypE in cellular processes are highly similar to those of CypA.

In this study, we found the interaction between CypE, a catalytic protein, and Runx2. Furthermore, we observed that CypE stimulates osteoblast differentiation and elucidated the underlying mechanisms regulating this process.

## 2. Materials and Methods

### 2.1. Cell Culture and Differentiation

Human embryonic kidney cells (HEK 293) and pre-myoblast cells (C2C12) were cultured in Dulbecco’s Modified Eagle Medium (DMEM) (#12100046; Gibco™, Carlsbad, CA, USA) supplemented with 10% fetal bovine serum (FBS) (S001-07; Welgene Inc., Daegu, Republic of Korea) and 1% antibiotic–antimycotics (#15240062; Gibco™). The cells were maintained at 37 °C in an environment with 5% CO_2_. As in previous studies, C2C12 cells are a commonly used cell line for studying osteogenesis [19]. Also, BMPs can convert the differentiation pathway of C2C12 cells into that of osteoblast lineage cells [20]. For osteoblast differentiation, after reaching full confluence (day 0), C2C12 cells were maintained in the differentiation medium (DMEM containing 2% FBS and stimulated with 50 ng/mL bone morphogenetic protein 4 (BMP4)) for 3 days.

### 2.2. Plasmids

The plasmid containing Myc-tagged Runx2 was cloned into the CMV promoter-derived mammalian expression vector (pCS4-3Myc). The full-length CypE (amino acids 1–314) and CypE mutations (CypE R191A and CypE W257A) were cloned into the pCS4-3HA vector. The CypE deletions (CypE 1–136, CypE 137–314, and CypE 187–314) were cloned into the pCS4-3Myc vector. The CypE gene was constructed in the pGEX-4T2 vector for the GST pull-down assay. All mutant and deletion plasmids were constructed using the oligonucleotide primer sets listed in Table 1.

### 2.3. Reagents

To identify the phosphorylation processes acting as upstream signaling mediators, various kinase inhibitors were employed in the study. The information can be found in Table 2.

### 2.4. Small Hairpin RNA (shRNA) Construction and Transfection

To transiently silence CypE, small hairpin RNA (shRNA) oligonucleotides were synthesized to target a 21-base pair (bp) sequence (AGG TTC TTC ATG CTG CAT TTA) of the mouse CypE gene. For the knockdown of *Runx2*, a 19-bp sequence (GT CCT ATG ACC AGT CTT AC) of the mouse Runx2 gene was used for transient silencing. The annealed oligonucleotides were ligated into the pSuper retro puro vectors (Oligoengine, Seattle, WA, USA). HEK 293 and C2C12 cells were transfected with both overexpression and knockdown plasmids using the polyethyleneimine (PEI) transient transfection method (Polysciences, Inc., Warrington, PA, USA).

### 2.5. Alkaline Phosphatase (ALP) Staining

To analyze the differentiation levels, ALP staining was used. The fully differentiated C2C12 cells were fixed in 4% paraformaldehyde, followed by being washed twice with phosphate-buffered saline (PBS). Finally, they were stained with 1-Step™ NBT/BCIP Substrate Solution (34042; Thermo Scientific, Waltham, MA, USA) at RT. The absorbance of ALP staining was measured at 480 nm using a microplate reader (Epoch; Bio-Tek Instruments, Winooski, VT, USA).

### 2.6. Luciferase Assay

To examine the promoter activity of osteogenic genes, the luciferase assay was performed using the luciferase reporter gene test kit (E1501; Promega, Madison, WI, USA) according to the manufacturer’s instructions. TriStar^2^ Multimode Reader apparatus was used to measure the luciferase intensity. All experiments were performed in triplicate.

### 2.7. Immunoblotting (IB) and Immunoprecipitation (IP) Two Wa

All protein samples were lysed using a lysis buffer containing 25 mM HEPES (pH 7.4), 150 mM NaCl, 1% NP-40, 0.25% sodium deoxycholic acid (Na-Doc), 10% glycerol, 25 mM NaF, 1 mM EDTA, and 1 mM Na_3_VO_4_, supplemented with protease inhibitors including 250 μM of PMSF, 10 μg/mL of leupeptin, 10 μg/mL of aprotinin, and 10 μg/mL of pepstatin. The extracted whole-cell lysate was separated using sodium dodecyl sulfate-polyacrylamide gel electrophoresis (SDS-PAGE) and transferred to polyvinylidene difluoride membranes (Immobilon-P; Millipore, Burlington, MA, USA). The membrane was blocked with 5% skim milk and incubated with the indicated primary antibodies overnight at 4 °C. Then, the membranes were incubated with horseradish peroxidase-conjugated (HPR) secondary antibodies (1:1000) for 1 h at RT. Finally, the membranes were visualized using Immobilon Western Chemiluminescent HRP Substrate (WBKLS0500, Millipore). Image acquisition was performed using the Amersham^TM^ ImageQuant^TM^ 800 system (GE Healthcare Life Sciences, Marlborough, MA, USA). The primary antibodies used are detailed in Table 3. For exogenous protein interaction, immunoprecipitation (IP) was employed. HEK 293 cells were transfected with overexpression plasmids, such as HA-CypE, Myc-Runx2, and Myc-Runx2 deletion mutants (N, R, C) for 48 h. Whole-cell lysates were collected using ice-lysis buffer, and an equal amount of cell lysate was incubated with appropriate antibodies (HA or Myc primary antibody) overnight and additionally incubated with Protein A Sepharose CL-4B for 1 h at 4 °C (#17096303; GE Healthcare Life Sciences). The protein–protein complex was eluted by heating at 100 °C and then detected using immunoblotting (IB).

### 2.8. Glutathione S-Transferase (GST) Pulldown Assay

To assess the interaction between Runx2 and CypE, a Glutathione S-transferase pulldown assay was performed as described previously [21]. Whole-cell lysates were prepared using a lysis buffer supplemented with protease inhibitors (described above) and incubated with Glutathione-Sepharose beads carrying GST or GST-Runx2/GST-CypE protein at 4 °C overnight. The bead–protein complex was then washed with the resuspension buffer (20 mM HEPES at pH 7.4, 120 mM NaCl, 10% glycerol, and 2 mM EDTA), eluted in SDS loading buffer, and analyzed through IB using the respective antibodies.

### 2.9. Reverse Transcription Followed by Quantitative Polymerase Chain Reaction (RT-qPCR)

The total RNA from C2C12 cells was isolated using a TRIzol reagent (TaKaRa, Tokyo, Japan). For each sample, 1 μg of RNA was converted to cDNA using the GoScript^TM^ Reverse Transcription System (Promega), following the manufacturer’s instructions. RT-qPCR was conducted using TB Green^®^ Premix Ex Taq™ (Tli RNaseH Plus) (TaKaRa) on a Bio-Rad real-time PCR system (CFX96) (Bio-Rad, Hercules, CA, USA). The mRNA expression levels were normalized to GAPDH, and each experiment was conducted in triplicate. The primer sequences for the target genes are detailed in Table 4.

### 2.10. Statistical Analysis

All experiments were conducted in triplicate, using different samples each time. The results were presented as the mean ± SD. Statistical comparisons between two groups were assessed using Student’s *t*-test. Differences among multiple groups were evaluated through one-way analysis of variance (ANOVA). Statistically significant differences were considered when *p* < 0.05.

## 3. Results

### 3.1. CypE Overexpression Promotes BMP4-Induced Osteoblast Differentiation in C2C12 Cells

**Figure 1:**
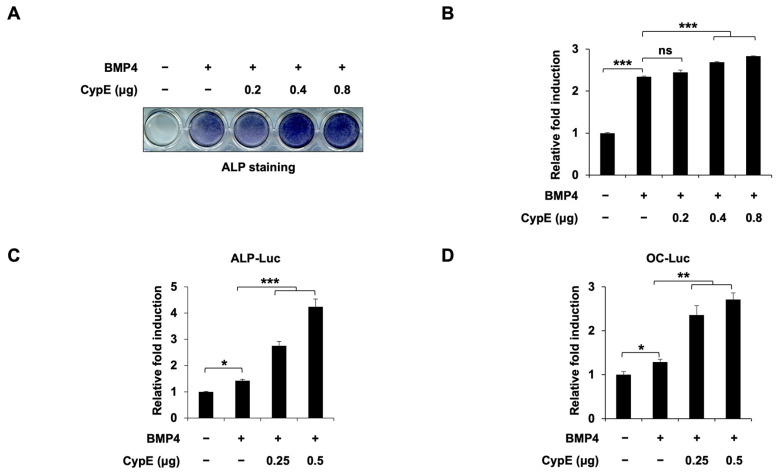
*CypE* overexpression promotes BMP4-induced osteoblast differentiation in C2C12 cells. C2C12 cells were transfected with a pCS4 empty vector or an increasing amount of CypE and treated with or without BMP4. (**A**,**B**) Representative image and quantification of ALP staining for CypE promotes BMP4-induced osteoblast differentiation. (**C**,**D**) The promoter activity of *ALP* and *OC* was measured via a luciferase assay. Results are presented as the mean ± SD, *n* = 3. * *p* < 0.05, ** *p* < 0.01, *** *p* < 0.001.

While the roles of other members of the cyclophilin family during osteoblast differentiation have been studied, the role of CypE in this process remains elusive. Alkaline phosphatase (ALP) is a well-known early marker for osteoblast differentiation [22]. To examine whether CypE could affect osteoblast differentiation, we overexpressed CypE and induced osteoblast differentiation using BMP4 in C2C12 cells and used ALP staining to assess differentiation levels. ALP staining was induced by BMP4 treatment in C2C12 cells, and *CypE* overexpression further increased BMP4-induced ALP staining and the mRNA expression of *ALP* (Figure 1A,B and Figure S2B). To confirm *CypE* overexpression after transfection, IB was conducted. The results showed that exogenous CypE expression was increased in a dose-dependent manner (Figure S1A). Consistently, *CypE* overexpression also enhanced the BMP4-induced transcriptional activity of osteogenic genes including *ALP* and osteocalcin (*OC*) (Figure 1C,D). These results suggest that CypE positively regulates osteoblast differentiation.

### 3.2. CypE Knockdown Suppresses BMP4-Induced Osteoblast Differentiation in C2C12 Cells

**Figure 2:**
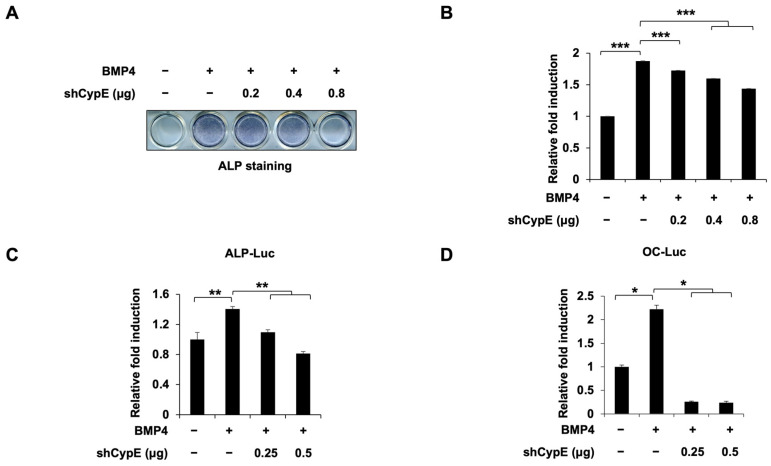
Knockdown of CypE attenuates BMP4-induced C2C12 cell differentiation. The C2C12 cells were transfected with an increasing amount of shCypE and a pSuper retro puro empty vector as a transfection control. After 24 h, C2C12 cells were treated with or without BMP4. (**A**,**B**) Representative image and quantification of ALP staining for CypE knockdown suppressing BMP4-induced osteoblast differentiation. (**C**,**D**) The promoter activity of *ALP* and *OC* was measured via a luciferase assay. Results are presented as the mean ± SD, *n* = 3. * *p* < 0.05, ** *p* < 0.01, *** *p* < 0.001.

Next, to confirm the positive effects of CypE on osteoblast differentiation, we investigated the impact of CypE knockdown on this process in C2C12 cells (Figures S1B and S2C). In contrast to *CypE* overexpression, *CypE* knockdown inhibited BMP4-induced ALP staining and the mRNA expression of *ALP* (Figure 2A,B and Figure S2D). Additionally, *CypE* knockdown inhibited the transcriptional activity of osteogenic genes including *ALP* and *OC* (Figure 2C,D). These results confirm our findings indicating that CypE plays a positive role in osteoblast differentiation.

### 3.3. CypE Interacts with Runx2 at the Runt and C-Terminal Domains

**Figure 3:**
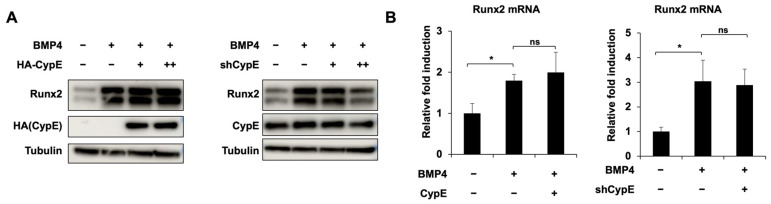

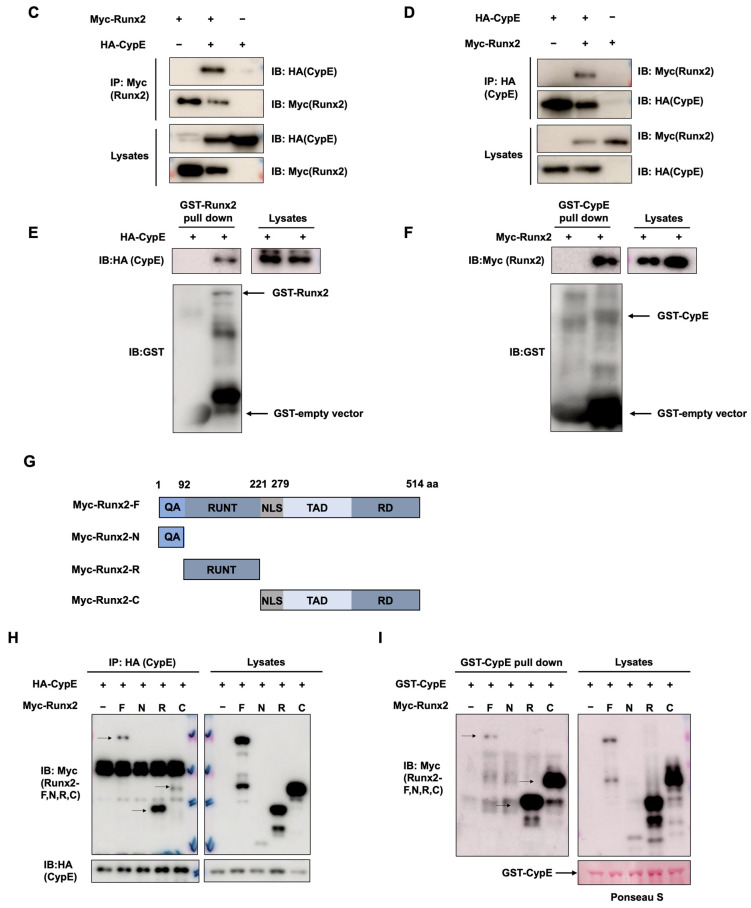
CypE interacts with Runx2 at the Runt and C-terminal domain. (**A**,**B**) C2C12 cells were transfected with indicated expression plasmids (HA-tagged empty vector, HA-CypE, pSuper empty vector, or shCypE) and treated with or without BMP4 for 48 h. Relative mRNA expression and protein expression of Runx2 were measured via RT-qPCR and IB. Tubulin was used as a loading control. Results are presented as the mean ± SD, *n* = 3. * *p* < 0.05. (**C**,**D**) HEK 293 cells were co-transfected with combinations of HA-CypE and Myc-Runx2 for 48 h. The protein interaction between Runx2 and CypE was confirmed by IP. (**E**,**F**) HEK 293 cells were transfected with HA-CypE or Myc-Runx2 for 48 h. The direct interaction between Runx2 and CypE was confirmed by GST-pull down assay. GST-empty vector as a negative control. (**G**–**I**) HEK 293 cells were transfected with HA-CypE, Myc-Runx2, and Myc-Runx2 (N, R, C) for 48 h. The interaction between Runx2 (F, N, R, C) and CypE was confirmed by IP and GST-pull down assay. –, empty vector; F, full length of Runx2; N, N-terminal domain; R, Runt homology domain; C, C-terminal domain.

To investigate the intracellular target of CypE, we used gain-of-function and loss-of-function experiments. We found that CypE affected the protein expression of Runx2, but not mRNA levels (Figure 3A,B). To further elucidate the relationship between Runx2 and CypE, we examined their potential interaction through IP and the GST pull-down assay. We found that CypE and Runx2 directly interacted with each other (Figure 3C–F). Next, we sought to determine which region of Runx2 was involved in this interaction. Following the methodology described previously [23], we employed a series of deletion mutants of Runx2, such as N-terminal domain (N), Runt homology domain (R), and C-terminal domain (C) (Figure 3G). Both overexpressed CypE and purified CypE interacted with the Runt and C-terminal domains of Runx2 (Figure 3H,I). These results suggest that the Runt and C-terminal domains of Runx2 are involved in the binding between Runx2 and CypE.

### 3.4. CypE Stimulates the Transcriptional Activity of Runx2

**Figure 4:**
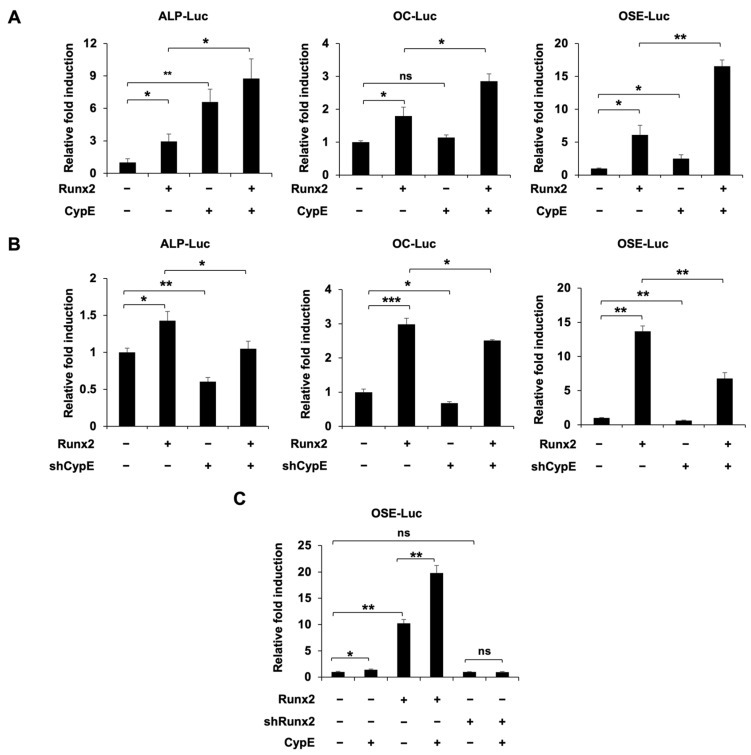
CypE stimulates the transcriptional activity of Runx2. (**A**,**B**) C2C12 cells were transfected with indicated expression plasmids (Runx2, CypE, shCypE, or empty vector (pCS4 or pSuper retro puro)) for 48 h. The promoter activity of *ALP*, *OC*, and *OSE* was measured via a luciferase assay. Results are presented as the mean ± SD, *n* = 3. * *p* < 0.05, ** *p* < 0.01, *** *p* < 0.001. (**C**) C2C12 cells were transfected with indicated expression plasmids (Runx2, shRunx2, CypE or pSuper retro puro empty vector) for 48 h. The promoter activity of *ALP*, *OC*, and *OSE* was measured via a luciferase assay. Results are presented as the mean ± SD, *n* = 3. * *p* < 0.05, ** *p* < 0.01.

As the interaction between Runx2 and CypE led us to consider how CypE targets Runx2, we aimed to explore the precise regulatory mechanism of CypE in osteoblasts. First, we performed a promoter activity assay using *ALP*, *OC*, and *osteoblast-specific cis-acting element* (*OSE*)-Luc, either separately or co-transfected with Runx2 and CypE. The promoter activity was increased upon *Runx2* overexpression and this activity was further enhanced by *CypE* overexpression (Figure 4A). Conversely, *CypE* knockdown suppressed the Runx2-induced promoter activity of *ALP*, *OC*, and *OSE* (Figure 4B). Next, we examined the effects of CypE in the absence of Runx2 to further understand the regulatory mechanisms. The knockdown efficiency of Runx2 was shown in Figure S4. Even though CypE enhanced Runx2-induced promoter activity, it failed to increase promoter activity when Runx2 was downregulated (Figure 4C). These results suggest that CypE exerts its regulatory effects on osteoblasts by stimulating the transcriptional activity of Runx2.

### 3.5. The Regulatory Effects of CypE Depend on Its PPIase Activity

**Figure 5:**
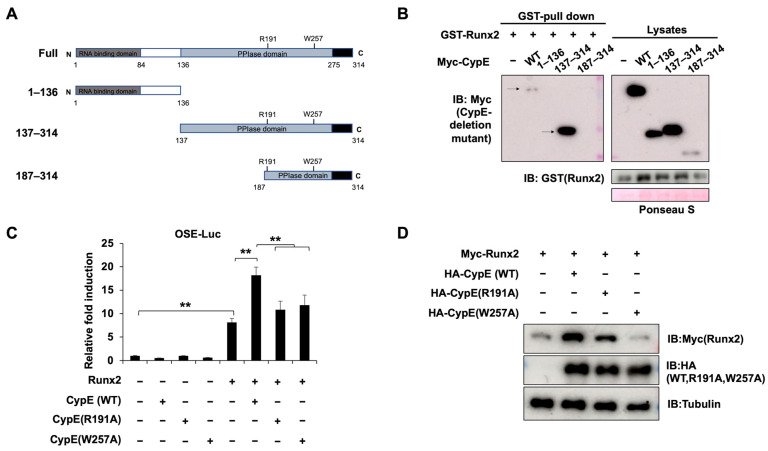
The regulatory effects of CypE are dependent on its PPIase activity. (**A**) The expression plasmid design of the CypE deletion. (**B**) HEK 293 cells were transfected with Myc-CypE and CypE deletion mutants for 48 h. The interaction between Runx2 and CypE or CypE deletion mutants was determined via the GST-pull down assay. (**C**) C2C12 cells were transfected with Runx2 and CypE or CypE point mutants for 48 h. The promoter activity of *OSE* was measured via a luciferase assay. Results are presented as the mean ± SD, *n* = 3. ** *p* < 0.01. (**D**) HEK 293 cells were transfected with Myc-Runx2 and HA-CypE or CypE point mutants for 48 h. The protein expressions of indicated genes were measured via IB. Tubulin was used as a loading control.

CypE is a member of the PPIase family and contains two functional domains: the nuclear binding domain at the N-terminus and the PPIase domain at the C-terminus [18,24]. To elucidate which functional domain interacts with Runx2, we constructed several deletion mutants of CypE (1–136 aa, 137–314 aa, 187–314 aa) (Figure 5A). Using the GST-pull down assay, we found that the primary binding region was the PPIase domain, specifically the residues 137–186 (Figure 5B). Previous research has identified two important sites within the PPIase domain of CypE: R191 and W257 [24,25]. To confirm the significance of CypE’s PPIase activity in its regulation of Runx2, we cloned the point mutants of CypE (R191A and W257A). As expected, both R191A and W257A failed to improve the transcriptional activity of Runx2 compared to the wild-type (Figure 5C). Furthermore, the protein levels of Runx2 were also suppressed by R191A and W257A (Figure 5D). These results indicate that the 137–186 domain of CypE is the primary region responsible for binding with Runx2, and the regulatory effects of CypE rely on its PPIase activity.

### 3.6. The Akt Signaling Pathway Is Involved in the positive Effect of CypE on Osteoblast Differentiation

**Figure 6:**
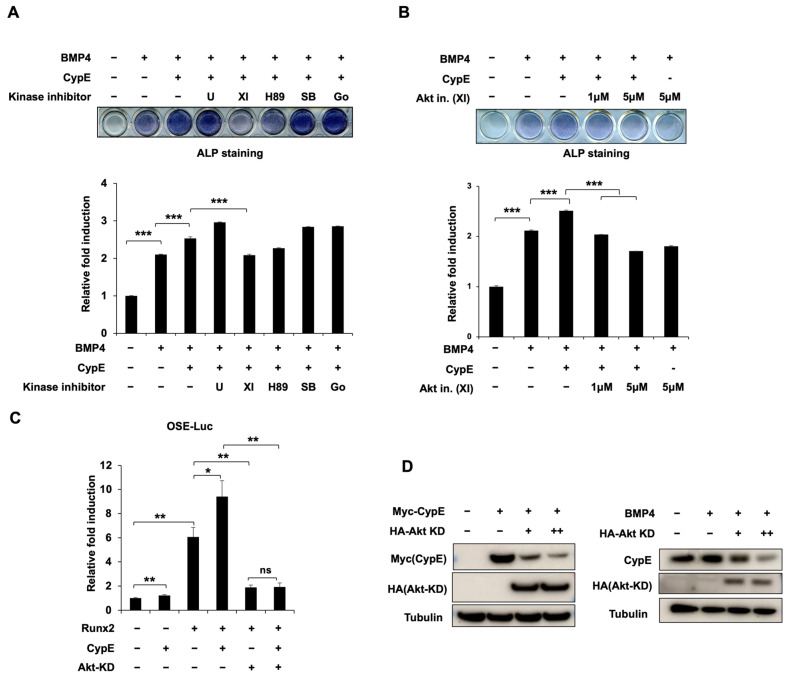
The Akt signaling pathway is involved in the positive action of CypE on osteoblast differentiation. (**A**,**B**) C2C12 cells were transfected with CypE and treated with indicated kinase inhibitors (10 µM of MEK inhibitor U0126 (U), 5 µM of PKA inhibitor (H89), 5 µM of PKC inhibitor Go6976 (Go), 5 µM of p38MAPK inhibitor SB203580 (SB), 5 µM of Akt inhibitor (XI)). After differentiation for 3 days, ALP staining was performed (top panel) and the density of ALP staining at 480 nm (bottom panel) was determined. Results are presented as the mean ± SD, *n* = 3. *** *p* < 0.001. (**C**) C2C12 cells were transfected with indicated expression plasmids (Runx2, Akt-KD, CypE, or empty vector) for 48 h. The promoter activity of *OSE* was measured via a luciferase assay. Results are presented as the mean ± SD, *n* = 3. * *p* < 0.05, ** *p* < 0.01. (**D**) HEK 293 and C2C12 cells were transfected with indicated expression plasmids for 48 h. The protein levels of CypE and Akt-KD were measured via IB. Tubulin was used as a loading control.

Various signaling pathways have important roles during osteoblast differentiation, including p38 MAPK [26], Akt [27], Wnt/β-catenin [28], etc. To examine which signaling pathway is involved in CypE-mediated osteoblast differentiation, we treated cells with various kinase inhibitors after CypE transfection. Among these kinase inhibitors, the Akt signaling inhibitor (XI) dramatically suppressed CypE-induced osteoblast differentiation (Figure 6A,B). To further elucidate the effects of Akt signaling on CypE-regulated osteoblast differentiation, we conducted additional experiments using a kinase-defective mutant form of Akt (Akt-KD). Akt-KD abolished the CypE-induced transcriptional activity of Runx2 (Figure 6C). Furthermore, Akt-KD also decreased the protein levels of exogenous and endogenous CypE (Figure 6D). These results suggest the Akt signaling pathway is involved in CypE-regulated osteoblast differentiation.

## 4. Discussion

In this study, we found a novel biological role of CypE in osteoblast differentiation. The overexpression of *CypE* resulted in the enhanced stimulation of BMP4-induced osteoblast differentiation. Also, we found that CypE alone (without BMP4) did not affect osteoblast differentiation, as shown in Figure S3. Conversely, the knockdown of *CypE* strongly attenuated BMP4-induced osteoblast differentiation. We first observed that CypE and Runx2 interacted with each other. Furthermore, CypE affected the transcriptional activity of Runx2 during osteogenesis. Finally, the Akt signaling pathway was found to be involved in CypE-mediated osteoblast differentiation.

Previous studies have reported that cyclophilins catalyze peptidyl-prolyl cis-trans isomerization, which involves various biological processes, including protein folding, signaling pathways, and biomolecular assembly [29,30,31]. Therefore, cyclophilins regulate biological processes in various disease models, including cancer [32] and immunodeficiency diseases [33]. CypA is the most abundant member and has dual roles in the regulation of bone formation and resorption [34]. CypB plays an important role in osteogenesis and bone development [35,36]. CypD also exerts essential effects on bone formation by regulating mitochondrial permeability transition pores and mitochondrial dysfunction [37]. Based on our previous research, we reported the detailed mechanism of how CypA promotes osteoblast differentiation by regulating the transcriptional activity of Runx2 [21]. Similarly, we observed that CypE also plays a positive role in osteoblast differentiation. It seems that the high degree of structural similarity at the cyclophilin-like domain between CypA and CypE may be responsible for this function. According to mutagenesis analysis, the PPIase activity of CypE is essential for its regulation of the transcriptional activity of Runx2. Furthermore, in contrast to CypA, which is widely expressed in C2C12 cells, we observed a dynamic expression pattern of CypE during C2C12 cell differentiation (Figure S2A). We hypothesize that the BMP4 signaling pathway can directly or indirectly affect the expression of CypE during osteogenesis. However, the exact regulatory mechanism requires further investigation. It is expected that as more roles of cyclophilin in osteoblast differentiation are elucidated, cyclophilin can be used as a biomarker or intracellular target. Moreover, the design of effective inhibitors or activators for the treatment of osteoporosis is of great significance.

Runx2 is a crucial transcription factor required for osteoblast differentiation and bone formation [38]. Clinical studies have revealed that a deficiency in Runx2 leads to cleidocranial dysplasia (CCD) [39]. Inhibiting Runx2 deacetylation using valproic acid [40] and MS-275 has shown potential for treating CCD [41]. Moreover, mouse experiments have demonstrated a close association between craniosynostosis and the regulatory mechanism of Runx2 [42]. More recently, Runx2 has been identified as a major cellular target for treating osteoporosis [43]. Therefore, targeting the expression and protein function of Runx2 constitutes an effective approach to regulating osteogenesis.

Various signaling pathways have been reported to affect the expression and protein function of Runx2 through the phosphorylation, acetylation, and ubiquitination of Runx2 after translation [44,45]. In our study, we found a relationship between Runx2 and CypE. Subsequently, to elucidate which signaling pathway is involved in CypE-regulated osteoblast differentiation, C2C12 cells were treated with diverse kinase inhibitors after CypE transfection. Among these inhibitors, the Akt signaling inhibitor (XI) exhibited a strong inhibitory effect. Furthermore, a kinase-defective mutant form of Akt (Akt-KD) also showed a significant inhibitory effect. Previous studies have shown that the Akt signaling pathway enhances Runx2 protein stability by regulating smurf2 functions during osteogenesis [46]. Specifically, the Akt signaling pathway controls Runx2 protein expression and activity through the direct phosphorylation of the Runt domain [47,48]. These findings suggest that the phosphorylation of Runx2 by the Akt signaling pathway is important for CypE-regulated osteoblast differentiation. Furthermore, Akt-KD affects the protein expression of CypE, emphasizing the importance of Akt signaling. Given the complexity of the signaling pathway, additional studies are needed to fully understand the relationship between the Akt signaling pathway and CypE. In our results, we also found that H89, a PKA signaling inhibitor, suppressed CypE-induced osteoblast differentiation, even though the suppression effect of H89 was lower than XI. In a previous study, PKA signaling showed positive effects on bone formation in vivo [49]. Also, PKA signaling modulates Runx2 and Osterix phosphorylation during osteoblast differentiation [50,51]. Therefore, PKA signaling might involve CypE-regulated processes. However, considering that the regulation of CypE by PKA signaling is not as strong as the Akt signaling, further studies are needed to clarify the minor regulation mechanism of CypE by PKA signaling.

## 5. Conclusions

Collectively, our data demonstrate that CypE is important for osteoblast differentiation and provide a concrete mechanism. In detail, CypE regulates osteoblast differentiation by increasing Runx2 transcriptional activity, and the Akt signaling pathway plays a crucial role in this process. Furthermore, the finding of CypE’s function in osteoblast differentiation suggests that the cyclophilin family could serve as a potential biomarker and cellular target.

## Figures and Tables

**Table 1:** The primers used for constructing the plasmids.

Target		Sequence
CypE_EcoR1	Forward	CGG AAT TCA ATG GCC ACC ACC AAG CGC
CypE_Xho1	Reverse	CCG CTC GAG CTA GAT GGT GTG GCT CTC
CypE_R191A	Forward	AGCAGCTTCCACGCCATCATCCCCCAGTT
CypE_R191A	Reverse	AACTGGGGGATGATGGCGTGGAAGCTGCTT
CypE_W257A	Forward	TGACAAGACAGACGCGCTGGATGGCAAGCA
CypE_W257A	Reverse	TGCTTGCCATCCAGCGCGTCTGTCTTGTCA
CypE_1–136	Reverse	CCG CTC GAG GGC CTT TTT AGC AAT GGG
CypE_137–314	Forward	CGG AAT TCA CGC TCA AAT CCT CAG GTG
CypE_187–314	Forward	CGG AAT TCA AGC AGC TTC CAC CGC ATC

**Table 2:** Kinase inhibitors used in the study.

Kinase Inhibitor	Catalog Number	Company
Mitogen-activated protein kinase (MEK) inhibitor (U1026)	662005	Calbiochem (San Diego, CA, USA)
Protein kinase A (PKA) inhibitor (H89)	371963	Calbiochem (San Diego, CA, USA)
Protein kinase C (PKC) inhibitor (Go6976)	345250	Calbiochem (San Diego, CA, USA)
Akt inhibitor (XI)	124028	Calbiochem (San Diego, CA, USA)
p38 MAPK kinase inhibitor (SB203580)	559386	Calbiochem (San Diego, CA, USA)

**Table 3:** Primary antibodies used in the study.

Target	Order #	Company	Dilution
CypE	sc-100700	Santa Cruz Biotechnology, Santa Cruz, CA, USA	1:500
Runx2	sc-390351	Santa Cruz Biotechnology, Santa Cruz, CA, USA	1:1000
α-Tubulin	sc-8035	Santa Cruz Biotechnology, Santa Cruz, CA, USA	1:1000
HA	12CA5	Roche Applied Science, Basel, Switzerland	1:1000
Myc	9E10	Roche Applied Science, Basel, Switzerland	1:1000

**Table 4:** The primer sequences for qPCR analysis.

Target	Sequence
m*CypE* forward	5′-ACACCGAGGGTTTGCTTTTG-3′
m*CypE* reverse	5′-GCTCAGATTCATTCATGTTGTCG-3′
m*ALP* forward	5′-ATC TTT GGT CTG GCT CCC ATG-3′
m*ALP* reverse	5′-TTT CCC GTT CAC CGT CCA C-3′
m*Runx2* forward	5′-CCT GAA CTC TGC ACC AAG TCC T-3′
m*Runx2* reverse	5′-TCA TCT GGC TCA GAT AGG AGG G-3′
*GAPDH* forward	5′-AGG TCG GTG TGA ACG GAT TTG-3′
*GAPDH* reverse	5′-GGG GTC GTT GAT GGC AAC A-3′

## Data Availability

The data presented in this study are available in the article.

## Supplementary Materials

The following supporting information can be downloaded at: https://www.mdpi.com/article/10.3390/cells12212549/s1, Figure S1. The expression of CypE in the overexpression and knockdown conditions. Figure S2. CypE has shown positive effects during osteoblast differentiation. Figure S3. CypE alone did not promote osteoblast differentiation. Figure S4. The knockdown efficiency of Runx2.
