# Supplementary material for: Cyclophilin E (CypE) Functions as a Positive Regulator in Osteoblast Differentiation by Regulating the Transcriptional Activity of Runx2
Source: Cells. 2023 Oct 31;12(21):2549. doi: 10.3390/cells12212549 (PMC10648996; doi:10.3390/cells12212549)
Supplement: Supplementary file 1 [file cells-12-02549-s001.zip › cells-2684481-supplementary.pdf]

Supplementary materials

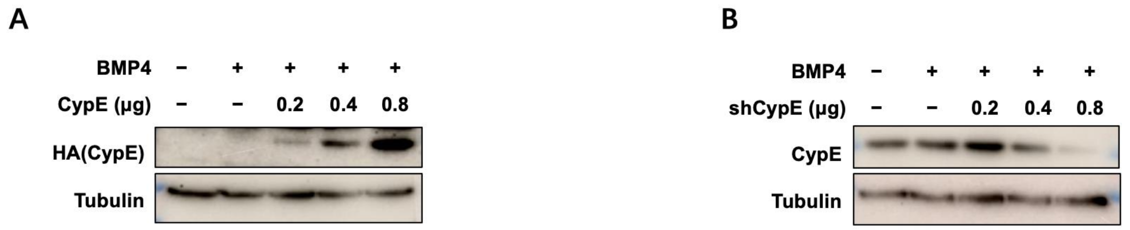

Figure S1. The expression of CypE in the overexpression and knockdown conditions. (A) C2C12 cells were transfected with pCS4 empty vector or increasing amounts of CypE, and treated with or without BMP4 for 3 days. (B) C2C12 cells were transfected with pSuper retro puro empty vector or increasing amounts of shCypE, and treated with or without BMP4 for 3 days. The protein expression of exogenous and endogenous CypE was performed by IB.

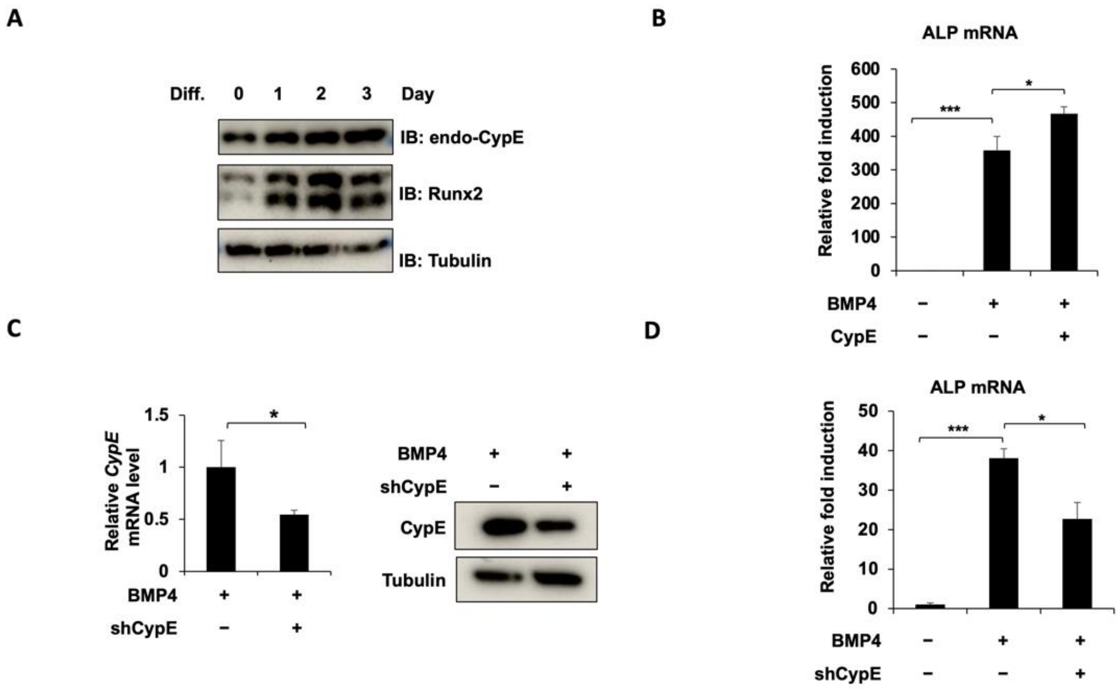

Figure S2. CypE has shown positive effects during osteoblast differentiation. (A) The protein expression of CypE and Runx2 during osteogenesis. (C) The knockdown efficiency of shCypE was performed by RT-qPCR and IB. Tubulin was used as a loading control. (B and D) C2C12 cells were transfected with indicated expression plasmids [CypE, shCypE, or empty vector(pCS4 or pSuper retro puro)] for 48 h. The mRNA expression of ALP was measured by RT-qPCR. Results are presented as the mean ± SD, n = 3. *$p < 0.05$, ***$p < 0.001$.

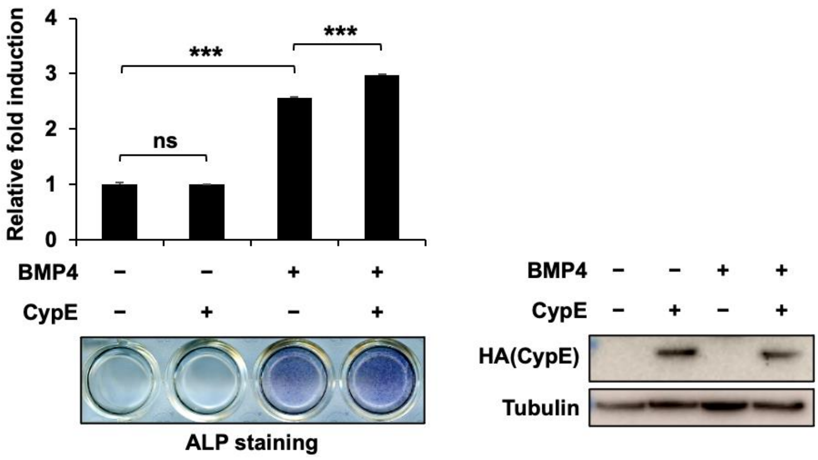

Figure S3. CypE alone did not promote osteoblast differentiation. C2C12 cells were transfected with or without CypE, and induced in the presence or absence of BMP4. Representative image and quantification of ALP staining (Left panel) and protein expression of CypE (Right panel) for CypE-regulated osteoblast differentiation. ns, no significant difference; *** $p < 0.001$.

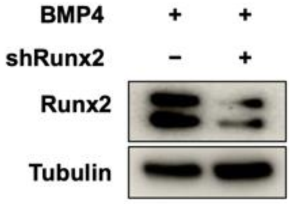

Figure S4. The knockdown efficiency of Runx2.C2C12 cells was transfected with pSuper empty vector or shRunx2 for 48 h. The protein expression of Runx2 was measured by IB. Tubulin was used as a loading control.
